# A Purr-Suasive Case for Sterilization: How Sterilizing Working Cats Supports Dairy Farmers’ Wellbeing, Improves Animal Welfare, and Benefits the Environment

**DOI:** 10.3390/ani15060766

**Published:** 2025-03-07

**Authors:** Caitlin Crawford, Jacquie Rand, Olivia Forge, Vanessa Rohlf, Pauleen Bennett, Rebekah Scotney

**Affiliations:** 1 Australian Pet Welfare Foundation, Kenmore, QLD 4069, Australia; jacquie@petwelfare.org.au; 2 School of Veterinary Science, The University of Queensland, Gatton, QLD 4343, Australia; rebekah.scotney@uq.edu.au; 3 The Far South Coast Branch, Animal Welfare League New South Wales, Bega, NSW 2550, Australia; oliviaforge@awlnsw.com.au; 4 School of Psychology and Public Health, La Trobe University, Bendigo, VIC 3552, Australia; v.rohlf@latrobe.edu.au (V.R.); pauleen.bennett@latrobe.edu.au (P.B.)

**Keywords:** free-roaming cats, One Welfare, dairy farms, rural cat management, sterilization, rodent control, Australia, cat cull impacts, Barn Cat Program, animal welfare

## Abstract

Rodents on farms are a substantial issue due to the damage they cause to produce and infrastructure, and the risk they pose to food safety. As a result, farmers need to control rodent populations around farms. In a previous study, farmers indicated a preference for working cats, stating that they are a more efficient, safer, and cheaper alternative to rodenticide baits, and they provide companionship. However, farmers’ options for managing cat populations are often limited to lethal methods because of time and financial constraints. Through semi-structured interviews, this study explored the impacts of lethal cat management methods on farmers, their experience with a free cat sterilization program, and their views on a Barn Cat Program, whereby healthy stray cats would be vaccinated, sterilized, and made available to farmers. Our results indicated that farmers had a relationship with the cats on their farms and that using lethal methods to manage these cats had a negative impact on farmers’ wellbeing. Farmers supported the free cat sterilization program, as it reduced the cat population and their impact on wildlife, improved cat behavior and welfare, and benefited farmers. Farmers also generally supported a Barn Cat Program and gave suggestions on how best to promote it to other farmers. Funding should be provided for cat sterilization programs, as they support farmers, who value cats for rodent control and companionship, improve cat behavior and welfare, and reduce working cat populations and their impact on wildlife.

## Simple Summary

Farmers must control rodent populations, as they cause substantial damage to machinery and pose health risks to humans and livestock. Working cats are often farmers’ preferred method of controlling rodent populations, as they are cheaper, safer, and more efficient than poison baits. However, without management, cat populations can increase, creating concern for their impact on wildlife and the environment, as well as for animal and human wellbeing. Farmers’ options for cat management are often limited by time and financial constraints, leading to the use of lethal methods. Our study aimed to investigate the impact of lethal cat management methods on farmers, their experiences with a free cat sterilization program, and their views on a Barn Cat Program, whereby healthy poorly socialized cats in shelters and pounds would be vaccinated, sterilized, and made available to farmers instead of being euthanized. The results of our study demonstrate that farmers had a relationship with working cats and that using lethal cat management methods negatively impacted their wellbeing. Farmers had a positive experience with the cat sterilization program, stating that it reduced the cat population and their impact on wildlife, improved cat behavior and welfare, and benefited farmers’ wellbeing. Farmers also supported the idea of a Barn Cat Program and gave suggestions for how best to promote it to other farmers. We recommend funding be provided to support farmers in managing cats by sterilization, as it improves cat welfare and behavior, reduces working cat populations and their impact on wildlife, and benefits farmers’ wellbeing.

## 1. Introduction

Rodent populations can have severe economic and health implications on farms, as they cause significant damage to infrastructure, produce, and crops and are vectors for spreading diseases such as leptospirosis, gastrointestinal infections, and rat-bite fever to humans and livestock [1,2]. Farmers commonly use rodenticides (lethal chemical agents, generally referred to as rat poisons) to control rodent populations [3]. However, rodenticides pose a safety risk to working dogs, children, and wildlife through primary and secondary poisoning, are expensive and time-consuming for farmers, and are becoming increasingly less effective due to rodenticide resistance [4,5,6,7,8,9,10,11]. Domestic cats (*Felis* catus) can offer an alternative form of rodent control. We have previously documented that Australian dairy farmers value having cats on farms for rodent control, stating they prevent thousands of dollars a year in costs associated with rodent damage to equipment and feed stores [5,12]. Because of their efficacy in preventing rodent damage to infrastructure, many farmers prefer having cats rather than using rodenticide baits, as working cats were viewed as more effective, cheaper, and safer for working dogs, children, pets, and wildlife [12]. These findings demonstrate that working cats play a vital role on farms; the value working cats hold for farmers should not be underestimated. Many animal welfare agencies in the USA and UK recognize cats’ value and implement working cat programs, where cats who are not suitable for rehoming in a family home are sterilized and placed on farms and businesses for rodent control [13,14,15,16,17,18,19]. In Australia, only a few programs exist [20], and concerns about wildlife, food safety, and cat-related nuisance complaints, such as urinating, defecating, and noise caused by fighting and females on heat, often lead to cats on farms and urban areas being culled rather than being managed humanely [21,22,23].

### 1.1. Current Cat Management Options for Farmers

Farms provide an abundant source of food for rodents, because of grain and seed stores, and a continuous supply of water and shelter [24,25]. This rodent population, in turn, provides a source of food for cats, contributing to a sustained population of cats. The numbers of cats around farm buildings are determined by the availability of food and, if the population is not controlled, can exceed 100 cats if there is sufficient feed for them [26]. However, the average number of working cats around farm buildings is 25 [27]. Although farmers value having some cats on the farm for rodent control [5,12], excessive numbers cause issues with fighting and soiling and concerns about the welfare of the cats, including that many kittens die from trauma and disease [28,29]. Options for managing working cat populations in Australia are limited. While it is legal for farmers to shoot and poison free-roaming cats and kittens to control cat populations, it is illegal in most states and territories for farmers to have cats sterilized without first taking ownership and paying registration fees and excess cat fees [30,31,32,33]. This may leave farmers with only lethal management options available to them, but having to kill healthy treatable animals has been shown to have negative psychological impacts. For example, participation in humane killing is linked with the presentation of symptoms consistent with post-traumatic stress and an increased risk of suicide in shelter staff [34,35,36]. Given that farmers have been shown to be at a higher risk than the general population for mental health issues and suicide [37,38,39,40] and demonstrate reluctance and negative emotions associated with euthanizing livestock [41,42], constraining farmers to use only lethal management for working cats may create negative emotions and add an additional risk factor for farmers’ mental health. Farmers who are reluctant to participate in lethal management may continue to let cat numbers increase. This could potentially lead to negative impacts on the welfare of those living and working on farms, cat welfare, and the environment.

### 1.2. Sterilization Programs

Though farmers value having cats on farms, there is a necessity to control free-roaming cat populations around farm buildings to reduce their impact on wildlife and food safety and for the cats’ welfare. Unfortunately, in Australia, current cat management options available to farmers may not be desirable or effective. Ovariohysterectomy/ovariectomy/orchiectomy (from here on referred to as sterilization) in cats has been shown to impact cat behavior positively, reducing fighting, activity levels, and roaming, whilst improving interactions with cat carers/owners and the body condition of cats [43,44,45,46,47]. These behavioral changes indicate that sterilization not only reduces cat populations but improves cat welfare and helps to decrease their potential impact on wildlife [5,28,45,48,49,50], whilst also being the favored strategy by the public [23,51]. With a lack of time for family and recreational activities, and financial pressures already being a risk factor for farmer’s mental health, there may also be barriers to controlling the cat population through sterilization [52,53]. Implementing a free cat sterilization program, which aims to assist farmers in caring for their working cats, could help to remove these barriers and support farmers in managing the working cat population in a way that benefits them and the environment. This strategy would encompass a One Welfare approach, which recognizes the interconnectedness of animal welfare, human wellbeing, and the environment [54]. Therefore, by reducing free-roaming working cat populations and improving their welfare, it would be expected that this would positively impact farmers’ wellbeing and protect the environment.

### 1.3. Gaps and Aims

Those living and working on farms have been shown to have a different relationship with the cats on their farm compared to people living in urban areas, with one study indicating that farmers see cats as valuable working animals rather than pets [5]. The literature on the relationship between farmers and their working animals is limited, as current research focuses on the human–animal bond between people and pets or the human–animal relationship between farm workers and livestock [55]. To this end, this study aims to address this gap by exploring relationships between farmers and their working cats.

Our study aimed to explore the experiences of dairy farmers managing multiple cats on their farms. The first part of this study focused on establishing the value of working cats to dairy farmers and their views on the Australian Tax Office classifying cats on farms as working cats [12]. The current paper addresses the second part of the study, focusing on three main aims. First, this study explored cat–farmer relationships. Second, farmers’ experiences before and after their involvement in a farm-cat management program, which primarily involved free cat sterilization, and their perception of its success was determined. Third, we investigated farmers’ views on a Barn Cat Program. With adequate resources, less socialized cats (cats who are fearful of unfamiliar humans) can often be rehomed as family pets; however, shelters and pounds frequently lack the space, time, and funding to properly socialize fearful and timid cats, leading to them being humanely killed. A Barn Cat Program would allow less socialized, healthy cats impounded in shelters and pounds, facing certain euthanasia, to be vaccinated, sterilized, and made available to farmers as working cats by animal welfare agencies at low cost. To ensure the retention of working cats enrolled in the Barn Cat Program, well-established protocols would be followed as outlined by a number of animal welfare organizations in the United States [56,57,58]. Our paper aims to provide insight into how cat management methods impact the social and psychological wellbeing of those living on farms as well as the welfare of the cats. Furthermore, this paper aims to help inform local government and welfare agencies of the impacts of assisting farmers with the non-lethal population management of cats they have on their farms.

## 2. Materials and Methods

### 2.1. Research Design

This paper reports on the second part of a larger study. The first part of the study focused on establishing the value of working cats to dairy farmers and their views on the Australian Tax Office classifying cats on farms as working cats [12]. The materials and methods of this phenomenological study were previously described in the companion paper [12] and are summarized here. A phenomenological approach allows researchers to understand a phenomenon from the perspective of the people involved [59]. Therefore, this methodological approach was an appropriate way to understand and explore the lived experience of dairy farmers who had cats on their farms and who had cats sterilized through one of two free sterilization programs (described below). The first author (CC) conducted semi-structured interviews to enable a deeper understanding of the value of cats to dairy farmers and the impact and outcomes of implementing an assistive-centered cat management strategy on dairy farmers.

### 2.2. Participants

The requirements to participate in this study stipulated that participants must be dairy farmers over the age of 18 years and residents of either Ipswich City, Queensland (QLD), or Bega Valley, New South Wales (NSW), have multiple cats on their farm, and have participated in one of two cat sterilization programs (described below). A total of 15 participants were recruited, 2 participants were recruited from the program in QLD, and the remaining were 13 recruited from the program in NSW. At the time of interviews, participants estimated between 3 and 60 cats resided on their property, with an estimated mean of 18 cats. Fourteen of the participants described themselves as being the owners of the cats (3–60 cats), and one participant stated that they did not own the cats (15 cats).

### 2.3. Procedure

Prior to commencing research, the University of Queensland Human Ethics Committee provided ethics approval (2024/HE000242). Purposive sampling [60] was used to reach farmers who had multiple cats on their farm and who had been involved with one of two cat sterilization programs. One program took place in QLD, Australia, whereby cat caregivers (semi-owners) and owners were provided with free cat sterilization (ovariohysterectomy/orchiectomy), microchipping, vaccination, endo- and ectoparasite control, and veterinary care for any issues affecting the welfare of the cats they were caring for. The program was initiated by the Australian Pet Welfare Foundation in association with the Royal Society for Prevention of Cruelty to Animals (RSPCA) Queensland, and the Animal Welfare League Queensland, with funding and in-kind support from multiple organizations, including the Fondation Brigitte Bardot for sterilizations, MSD Animal Health for vaccinations and parasite control, Central Animal Records for registration of microchips, and Neighborhood Cats USA for funding and expert advice. Cats participating in this program were categorized as restricted matter and were, therefore, permitted to be sterilized, microchipped and ear-tipped, and returned to their original location without being legally registered to an owner (approved by the Queensland Government under a Department of Agriculture and Fisheries Scientific Research Permit No. PRID000825). Each cat was registered as a ‘Community Cat’, with the Australian Pet Welfare Foundation phone number listed as the secondary contact when there was no owner registered on the microchip. The second program, ‘The Far South Coast Dairy Cat Project’, took place in Bega Valley, NSW, Australia. In this program, dairy farmers in Bega Valley, NSW, were provided with free cat sterilization (ovariohysterectomy/orchiectomy) and were assisted in rehoming unwanted kittens. Any ill or injured cats or kittens were humanely euthanized as were cats in excess of the farmers’ needs. This program was undertaken by the Animal Welfare League New South Wales with further funding and support from the South East Local Land Services, the Far South Coast Landcare Association, and local Bega veterinary practices. Nine farms participated in the study with a total of 392 working cats managed in the program (Appendix A).

Eligible farmers were invited to participate in this study via phone and email. Those who had participated in the program in QLD were contacted by the researcher who conducted the interviews (CC), and those who participated in the program in NSW were initially contacted by the program coordinator. Those who were interested in participating in the study were emailed or given a printed version of a participant information sheet and consent form. This document informed individuals that participation in the study was voluntary and confidential and that they did not have to answer any questions they did not feel comfortable with and could withdraw at any time.

Fifteen participants were recruited in total, from nine farms. Thirteen interviews took place between 5 April 2024 and 8 May 2024 (program participation occurred between September 2020 and March 2024). In two of the interviews, there were two participants, the remaining interviews had one participant. The data were collected using semi-structured in-depth interviews. Nine of the interviews were conducted in person, with the remaining four were conducted via phone. All interviews were voice recorded using an Olympus Voice Recorder VN-541PC.

The interviews lasted between 29 min and 62 min (average 43 min). During the interviews, participants were asked what the cats’ purpose was on the farm and if there were any benefits to their presence; what the farmer’s experiences were without having cats on the farm (if relevant); what their relationship with the cats was like; about their experiences before and after becoming involved with the sterilization program; what their views were on cats being classified as working cats by the ATO and, if supportive of this classification, how they might justify this. Finally, they were asked about their views on a Barn Cat Program (where healthy stray cats that are difficult to place in a home and are at risk of being euthanized would be vaccinated and sterilized and made available to farmers as working cats) and how this could best be promoted to farmers. Once all interviews were completed, 11 were transcribed by the first author (CC). The remaining two interviews, which had two interviewees each, were transcribed by a professional transcription service (Pacific Transcription Pty Ltd., Brisbane, Australia).

Thematic analysis was used to analyze the transcripts to search for recurring words or units of meaning, which were then organized into groups and themes [61]. To do this, the transcripts were coded, and these codes were then organized into corresponding themes. The research team discussed the data interpretation, and the themes were reviewed until the research team reached a consensus. In order to protect the privacy and confidentiality of participants, animal names were changed, and human names were excluded. Statistical analysis was conducted using SPSS version 29. Pearson’s correlation was implemented to identify the association between initial cat populations on farms and ‘reason for euthanasia’ variables as well as the association between the total percentage of cats euthanized and the percentage euthanized due to excess numbers. 

Data from this study on the value of working cats to dairy farmers and their view on the Australian Tax Office classifying cats on farms as working cats has been reported [12]. This current report presents results related to three additional aims: firstly, working cat–farmer relationships; secondly, farmers’ experiences before and after their involvement in the program and their perception of its success; thirdly, farmers’ views on a Barn Cat Program and how this could best be promoted to farmers.

## 3. Results

Analyses of the interview transcripts for this current report revealed 10 primary themes and several sub-themes. Four themes arose from questions focusing on dairy farmers’ experiences prior to becoming involved in a working cat sterilization program, a further four themes arose from questions focusing on farmers’ experiences after becoming involved in the program, and two themes arose from questions regarding farmers’ relationships with cats and their views on a Barn Cat Program. These primary themes and sub-themes are discussed with context examples in the sections below.

### 3.1. Farmers’ Relationships with Cats

Farmers’ relationships with working cats on the farm varied. Some individuals compared cats to livestock, and others stated that they loved them. All but one participant considered that they owned the cats on the farm and, when farmers discussed the relationship they had with the cats on the farm, most indicated they had a bond with some of the cats. One farmer stated they had a few favorites but compared their general relationship with the cats as similar to that of the cows. Farmers discussed the cats having a positive impact on their mood and the enjoyment of interacting with them. Some farmers stated that the cats were part of the farm or the family.

*‘Oh, I love them. They’re great. You’ve seen them. They curl up your leg and sleep with the calves, yeah, they’re good. Kids especially love them. They love coming down here and scooping all the kittens up and going for walks. I love the farm cats.’*

*‘Always a big smile when you see Freddy walk in, pretty sure he makes everybody smile like ohh, and then he gets a little handful of kibbles. But Dad can be in a cranky mood and he’ll come in and be cranky at us, and then he’ll see the cats and just be talking to them like they were the best things in the world.’*

*‘There’s Mable, the mother of this one, she was quite friendly, and we had another tomcat called Frank, and he was very friendly. He’s the father of this one here. They were the ones we’re very close to, but the rest were just like… probably like a cow or something, […] you feed them and yeah, yeah, just part of the farm.’*

*‘I find them [the cats] entertaining, really, all their little personalities.’*

All the farmers that were interviewed provided cats with milk, most farms gave additional cat or dog food, and some farms provided veterinary care for all or some of the cats.

*‘I make sure they’ve got feed, backpackers feed them or [my husband] feeds them. They’ve got turkey necks in the fridge, so every day they get a turkey neck.’*

*‘If we ran out of biscuits my mum would even cook up… she’d boil rice and put tuna in it because, like, we live out of town, so it might be a week before someone gets to Bega. So the cats would be there all meowing, so Mum would cook up this big pot of rice and tuna for them.’*

*‘Actually, I’ve just [brought] one [a cat] back home here now. She got ill and [I] took her to the vets, they couldn’t really diagnose what’s wrong with her. I think she’s had a mini stroke. Yeah, so I didn’t feel it was good for her back at the dairy.’*

### 3.2. Prior to Sterilization

Farmers discussed a number of issues they had prior to cats being sterilized through the free program, including nuisance behaviors, poor cat welfare, and concern for the cats’ impact on wildlife. Additionally, options that were available to them and barriers to managing cat populations prior to becoming involved with the sterilization program were discussed. Interestingly, farmers also relayed the impact the issues had on them and their wellbeing. These themes are provided with context examples below.

#### 3.2.1. Issues with Cat Behavior and Welfare

When discussing the behavior of the cats prior to sterilization, farmers indicated that there were a number of issues, primarily with fighting and defecation. Specifically, fighting caused a lot of noise, and the defecation and urination led to bad smells and mess around the dairy.

*‘Oh look it was… it was messy, it wasn’t nice, you know, there’d be a lot of cat shit everywhere.’*

*‘Oh, they [the cats] used to bloody pee all over everything in the shed. That’s not good, then there’s rust.’*

*‘Every time a cat was on heat though, they would mate. All the boys, […] like the two brothers would fight, or there was another male that came in and they would fight.’*

Often these behaviors and the increasing population size compromised cat welfare. Farmers discussed that prior to sterilization cats had poor body conditions and poor health due to viruses, such as cat flu. Additionally, accidents involving machinery, or injuries due to fighting often led to cats having quite severe wounds or injuries which farmers often could not treat because they were unable to handle the cats. These issues often lead to the death of affected cats with some farmers indicating that dead cats were found daily around the farm. Some farmers also discussed instances of infanticide; they would often find dead or ‘half-eaten’ kittens around the dairy.

*‘The other issue that we had was cats just dying from cat flu all the time, you’d have dead cat carcasses on the road. So it’d be a constant like you’re picking up each day, we’re picking up a dead cat.’*

*‘I just remember in winter, [if] we had a wet, cold day in winter, and you’d walk in and it would be early in the morning. [I would] see these kittens newborn kittens lying on the floor on the wet concrete floor. So, you’d run around and find an old rag and just sit them on there while you go and work. You come back and think, right, we gotta do something, get rid of these kittens. And then you’d come back and they’d be half eaten and then you think, oh, that’s even worse. Like, it just wasn’t a pleasant atmosphere at all.’*

*‘Look, you know, there’s a lot of, you know, eyeballs they’ve been scratched and infected cats and stuff like that. So, you know, there’s yeah, cats getting wounded from fights, […] they get infected legs or whatever, from their bites and stuff like that.’*

Of the nine farms that participated in the study, a total of 392 working cats were managed in the programs. Approximately 52% were sterilized and returned, 21.7% were sterilized and rehomed, and 25.5% were humanely euthanized, with 16.3% of those euthanized for health or welfare reasons and 9.2% because there were too many of them for the farmer’s needs. This reduced total working cat numbers on participating farms by 47.2% (see Appendix A). There was a significant positive association between the initial cat population and the total number euthanized (*p* ≤ 0.001) and the total initial population and the number (*p* ≤ 0.001) and percentage (*p* = 0.004) of cats euthanized for welfare reasons. There was also a significant positive association between the total percentage of cats euthanized and the percentage of cats euthanized due to excess numbers (*p* = 0.001).

#### 3.2.2. Concern for Cats’ Impact on the Environment

Farmers indicated that, prior to the sterilization program, they had concerns about the cat’s impact on wildlife due to witnessing hunting behaviors and finding dead wildlife around the dairy. Some farmers reported an awareness of the potential impact of increasing cat numbers on wildlife and stated this was not something they wanted. Regardless, a trade-off between controlling the rodent population and limiting the impact of cats on wildlife was discussed.

*‘It was on my mind that we were getting too many cats, they were sort of just increased each year and I do like the birds […] They were sort of hunting the birds and if they had kept going, […] it just would have got really harmful.’*

*‘Look when the population got big, the cats would move out into the environment, like some cats would move away and just go feral in the wild, and I don’t like feral cats in the wild. I like them to be, you know, domesticated or semi-domesticated. I don’t like killing wildlife.’*

*‘Yeah, but the fact that they [the cats were] bringing all the Galahs and parrots and whatever they get their hands on into the thing [the milking shed] like—jeez I got sick of that.’*

#### 3.2.3. Limited Cat Management Options

Some farmers were not managing cat numbers prior to their involvement in the sterilization program and mentioned having limited options. Specifically, if the situation had continued as it was, they would have used lethal strategies to manage the cat population. Conversely, several other farmers were already attempting to manage the cat population using lethal management methods, such as shooting and blunt force trauma to kill kittens. However, farmers stated that this was ineffective and indicated their reluctance to use these lethal management methods. A small number of farmers explained that they were trying to sterilize some of the cats, and others mentioned that they were letting the situation run its course without intervention.

*‘Finding the kittens and euthanizing the kittens as soon as we got them. […] Yeah, early, like, before their eyes were open.’*

*‘Yeah, using a cat trap and a bit of feed in there and just done… it’ll probably take me three to four days just over a period of time while I’m working and just catch one and take it around the corner and yeah, and shoot it. […] One day there I had four bags, big bags of cats to take away.’*

*‘I just trap them, I just take around the corner of the dairy where there’s a lot of noise with the vacuum pump so that no other cats could hear or anyone could hear what’s going on. The cat was distracted by the noise and they do still a couple laps in the trap. [You] slide the rifle in and just pop, take them out, put them in a bag, reset the trap, process over and over again till you sort of got the numbers down.’*

*‘We were getting any males desexed [sterilized] that we could catch and take in to the vets. We haven’t done anything with the females, especially not the younger kittens that were around.’*

When farmers were asked if they had considered sterilizing the cats as a management option, barriers such as cost, time, and difficulty were discussed. Farmers also revealed a lack of understanding about the situation, not knowing what they could do, and a feeling of a lack of control.

*‘You know, […] even catching the cats, that sort of thing, we had no idea. Yeah, yeah, which is one of those things you just sort of you knew was there, but [we] didn’t know how to attack it. […] What the action plan was to actually bring it under control.’*

*‘I suppose there wasn’t any opinion on how to manage the cats because there didn’t seem to be any options. You weren’t going to catch them and you weren’t going to go and pay vet bills to get them desexed not knowing whether they’re going to hang around or not afterward.’*

*‘The trouble is on the farm, we don’t have time for that stuff. You know, and for someone to do [it], you know, when I say someone, either [my brother or myself], to do what [the program coordinator] was doing [trapping, transporting and sterilizing cats], we would have had to have taken four or five hours out of our day.’*

During the interviews, some farmers indicated that the farming community had negative views of animal welfare agencies and a distrust of authorities. Furthermore, there were reports of feeling dubious about the sterilization program as farmers were unsure of the intentions of the program and outcomes for the cats. These negative perceptions held by the farming community and their distrust of authorities appear to be further potential barriers to seeking help with controlling cat populations.

*‘She just came and had a chat and had a look around and… and Dad has always been not to euthanize, he thought it was a euthanization program and so he was not on board.’*

*‘Unfortunately, yeah, there’s that stigma, and [the] Animal Welfare League will be just thrown in with PETA and all that sort of stuff and… and people have their back up straight away. But I think if we can use testimonies where farmers, the more farmers that have done it will probably help stop that.’*

*‘I was a bit dubious of [the program coordinator] coming in the beginning, because I didn’t know her and like she [promoted] it as a good cat program and getting them in, but you don’t know what it’s sort of like. You know, desex them [and] take most of your cats and then when you want [some], you end up with none and then you got rats and mice problems.’*

#### 3.2.4. Negative Impact on Farmers

When discussing the cat’s behavior and welfare prior to sterilization, farmers indicated that the situation was negatively impacting their wellbeing. Specifically, knowing there was a growing number of cats on the farm with no understanding of how best to deal with them was stressful. Moreover, farmers reported negative feelings related to regularly seeing sick and injured cats and kittens and having no ability to help them. One farmer said they felt embarrassed that they could not provide appropriate care for the cats.

*‘Oh, you feel shit when you look at a sick cat. It’s like, oh, like your mood changes when you see a sick animal. […] But you can’t help it, like you just want to help it, but you’re like I can’t catch you, so it’s, you know, good luck, mate.’*

*‘I think the hardest part was all the kittens. You just have all these kittens being born everywhere, anywhere and everywhere the cats could have them, and that was really stressful. That was, yeah, it was heartbreaking.’*

*‘Yeah, that’s not very good because you knew that one of them [the cats] was going to end up getting hurt. […] if we saw it happen [the cats fighting], we tried to stop [it] but yeah, obviously we couldn’t. We couldn’t [if] they weren’t near the dairy or anything like that.’*

Farmers also experienced negative impacts relating to the use of lethal management options, with many highlighting their dislike of the humane killing of animals. However, several stated that this was part of being a farmer, so they ‘just got on with it’. Farmers who were yet to start using lethal management methods revealed that it was something they were worrying about having to do and that they were delaying having to do it.

*‘I just, you know, say a little silent prayer to each one every time it’s a cow or a dog or whatever I’ve got to euthanize is, just a silent little prayer and just hope they forgive me for what I’m about to do and… and just do it and just yeah. I guess I’ve done it so long over the years I just turn off a little bit and… but yeah, still, it’s not a job I enjoy.’*

*‘The shooting part was pretty stressful on the cat and probably me as well, because you’d have to trap them and you’d have to settle them down in the trap and, yeah, pull the trigger.’*

*‘Well, I’ve been a farmer all my life, so you know, I’ve had animals, and you just have to compartmentalize that, you just have to go and do it. You do it and you put the gun away and you just move on. […] Yeah, otherwise you…you don’t think about it too much because you don’t want to.’*

*‘Well, that was the worry—what you’d have to do with them [the cats]. Yeah. Then you put it off for the next day, and the next day.’*

### 3.3. After Sterilization

When discussing their experience after participating in a working cat sterilization program, farmers reported an improvement in cat behavior and welfare and a perceived reduction in the cats’ impact on wildlife. Farmers spoke positively about the program, indicating that being involved with the working cat sterilization program had a positive impact on their emotional and psychological wellbeing. These themes are provided with context examples below.

#### 3.3.1. Improved Cat Behavior and Welfare

When asked about their behavior since the cats had been sterilized, farmers indicated seeing an improvement, including a reduction in fighting and soiling, and that the cats appeared more calm and friendly. Furthermore, it was reported that cat populations had stabilized.

*‘They just seem to be a lot healthier and fatter and oh my gosh, the biggest change has been how friendly they all are, they’re all friendly now.’*

*‘We do like them [the cats] better because there’s no fighting and the numbers aren’t growing, so we just accept our batch of 11, we’ve got there and look after them.’*

*‘Well, previous to the sterilization, the cats were very, very busy. Very, very cagey of us. We would have been able to pat maybe two of them previous to sterilization. I reckon three months after [they were] desexed, we could have patted ten of them.’*

Additionally, farmers discussed an improvement in cat welfare, stating that the body condition of the cats had improved, and the number of cats dying or being euthanized from injury or disease had reduced. Farmers also indicated that cats appeared healthier.

*‘I definitely haven’t had to put as many cats down from illness or injury since they’ve been desexed, as [I] was before.’*

*‘So, I’d have to say the health dramatically improved […] A surprising level, yeah. […] I would have thought, you know, some kind of improvement, but I wouldn’t have expected what we’ve seen.’*

*‘I think they [the cats] all seem much happier and healthier, and they just put on weight easier.’*

#### 3.3.2. Reduction in the Impact of Cats on the Environment

Farmers indicated a perceived reduction in the cats’ impact on native wildlife. Many reported seeing less evidence of dead wildlife and an increase in sightings of native wildlife around the farm. Additionally, a perceived reduction in cats’ home ranges was also reported.

*‘I’m not saying they don’t go and catch the occasional bird, they do, but I think the effect on the wildlife has drastically decreased.’*

*‘They used to bring birds in here in the office and you’re coming in [and] there’d be feathers everywhere, so we’ve noticed there’s none of that now. Which is good so they don’t seem to be roaming as feral as they used to be.’*

*‘[Prior to sterilization] you’d see them [the cats] down in the gully or in the paddocks and they’d be out hunting, but now, I swear they just kind of hang around the dairy area.’*

#### 3.3.3. Positive Views on the Working Cat Sterilization Program

All farmers perceived the program as being successful, with the average working cat population on farms decreasing by almost half (47.2%). Farmers conveyed a positive view of the sterilization program they were involved with, using words such as ‘excellent’, ‘awesome’, and ‘fantastic’. Many farmers reported that sterilization was the best strategy to manage the cats. Several farmers mentioned the rapport they had built with program staff and expressed that they would recommend the program to other farmers. Furthermore, they expressed hope that the program would continue and expand.

*‘It’s been an awesome program and if they can roll it out in other areas, then that’s great. And I do feel like that would also help the feral cat population in Australia as well.’*

*‘If the funding keeps going, well I think we’ll definitely keep going, we’ll just give [the program coordinator] a ring say look you bring traps, we’ve got a couple of cats here. We want cats, but you know it’s better than going back to what we used to do.’*

*‘This sterilization programs have been great, especially for our area. Once I got in contact with [the program coordinator] […] we grew kinda a greater connection together, and in the end, I started connecting her with a lot of dairy farms around the place. So a lot of the dairy farms around the place have had sterilization done to their cats, and I think that’s been a godsend for them that this program was available to us.’*

Farmers relayed how having the support of staff working for the program impacted its success. Because of the nature of farming, farmers indicated that they often lacked the time and resources to deal with the cats in the way they wanted and having support helped them take control of the situation.

*‘I suppose the option from what [the program] offered, was a form of starting to take control of the cats.’*

*‘I think this program has been excellent. When you’ve got somebody else that can come in and help you, and to liaise with the vets. And just have a day where they [cats] all go and [get] it all done at the same time, it was really seamless.’*

*‘As someone like myself, that’s a small business owner, that you know is very, very busy running a dairy farm and growing crops and feeding cattle and feeding calves and all the stuff that goes with it, and it’s seven days a week. To have someone to do a program and […] to do all the heavy lifting as far as the sterilization goes, that was a brilliant way to go.’*

Whilst the overall view of the cat sterilization program was positive, some farmers showed concern about replacing cats after all their cats had been sterilized, specifically highlighting their worry about the cost of buying sterilized cats from animal welfare organizations.

*‘Well, it’s interesting because now that they’re all desexed and we know that there’s no replacement plan going on, I am thinking in the future, how am I gonna… what am I gonna do when these cats eventually pass all away?’*

*‘My only concern about this program is, and obviously it’s not a concern at the moment because [the program coordinator’s] got more that she’s bringing in, is that to replace the cats it’s quite expensive, to buy desexed cats, you know. […] And that’s my only concern. I think everybody should participate in the program, but there should be some measure whereby we can replace these cats without huge expense of getting them from the Animal Welfare League at $800 a cat.’*

*‘I just don’t know what’s going to happen if you get them all desexed and then they start getting too old and they start dying off. Then we’ll be out. We won’t have a cat. We’ll have to go and buy some, or something.’*

#### 3.3.4. Positive Impact on Farmers

When discussing improvements in cat behavior and welfare, farmers indicated that this also had a positive impact on their wellbeing. Farmers stated that they no longer had to worry about the situation regarding the cat population and that the positive change in the cats created a more pleasant environment around the dairy.

*‘Not having to dispose of cats all the time is a lot better, yeah, a lot better.’*

*‘I think it’s just more relaxed, it’s one less thing that you have to worry about.’*

*‘The sterilization of the cats totally changed the nature of the cats, and therefore, I guess gave us a little bit of warm and fuzzy that we can now touch these cats and give a bit of a pat.’*

Additionally, farmers revealed that being involved in the farm-cat sterilization program created feelings of achievement and pride when comparing the situation before and after sterilization of the cats, providing evidence of the positive impact on farmers’ wellbeing.

*‘Good, yeah. You achieved something.’*

*‘And that was so great that we could rehome some of them, the ones that were young and friendly, that were able to be rehomed, that was really rewarding as well.’*

*‘Well, obviously you like them to be healthy and yeah, makes you feel good to know you’ve made their lives better, and they’re helping you and the whole thing’s, yeah, better.’*

### 3.4. Positive Views on the Barn Cat Program

Most farmers viewed the Barn Cat Program in a positive light, indicating that it was a better option than culling them, that it would prevent cat overpopulation on farms, and that there was a demand for the cats. However, some farmers had reservations, stating that cats had to be semi-domesticated for them to be successful barn cats and that farmers should have to pay a small fee for the cats to ensure they would be taken care of.

*‘The farmer would need to do something. You couldn’t just—they [farmers] would all go, yes, that’d be great, vaccinated, flea [treatment], desexed, yes, we’ll have 10, but then they need to be accountable for that animal’s life, I think, they can’t just see it as a free thing. Do you know what I mean? Maybe it’s not free. Maybe they’re $25 each or something. Maybe they have to check in every year. Maybe they need their microchip scanned every year at the farm. Maybe someone needs to go and do that.’*

*‘Yes, no, definitely, cause like I just said before, some farms were asking for cats, but the big thing for them is they didn’t want them to have litters. […] So it’s like it’s one of those things where there is a demand to have cats on farm, but you don’t want to have the situation that we were in, where you’ve got too many to handle.’*

*‘I reckon that’s a great idea because when we were looking for cats, we were struggling to find the first couple of cats that we needed. So for a program to be out there where the farmers can take these ready-made cats that are desexed, vaccinated and everything’s been done for them, and they’re ready to go into a working program. I think that’s a great idea to save euthanizing the cats.’*

When asked how the Barn Cat Program could best be promoted, many farmers indicated that word of mouth or through farming channels would be most effective. Farmers also stated that information regarding the benefits of having sterilized cats around the farm should be provided and that promoting the program would be more successful if it was coming from other farmers.

*‘So I think if… if that was advertised through the farming networks, I think there would be an uptake. […] You know quite often, you see people on the farm pages saying does anyone get any kittens or something like that, we need kittens for their hay shed or something like that. So, I think there would probably an uptake if the option was there for a desexed cat, treated, ready to go. I don’t think that’d be [an issue]. [We’re] probably not against paying a small fee for that as well.’*

*‘I think probably case studies to start with. Where you have got cats like that on a farm and the difference that they’re making and then definitely the cost saving and those things that we talked about before. Because if people can see that there’s money in it for them, and they don’t really have to do much, then it’s a pretty easy sell.’*

*‘Yeah, word of mouth would be the first one. Yeah, for sure, because like even, our workers have probably said to their other mates, oh do you know where to get a cat or two and doesn’t take long you know.’*

## 4. Discussion

A qualitative analysis was conducted with dairy farmers who had multiple cats on their farms and who had participated in an assistive-centered, working cat sterilization program. This study aimed to report on three aims: firstly, working cat–farmer relationships; secondly, farmers’ experiences before and after their involvement in the program and their perception of its success; thirdly, farmers’ views on a Barn Cat Program and how this could best be promoted to farmers. The key findings of our research were the positive relationships farmers had with their working cats, the issues relating to problematic cat behavior and poor welfare, concerns about the cats’ impact on the environment, and the limited available management options encountered prior to involvement with the program. Importantly, the farmers perceived that these issues improved after engaging with the working cat sterilization program, and this had a positive impact on farmer wellbeing, the welfare of the cats, and the environment. Additionally, we found that farmers generally supported the idea of a Barn Cat Program and provided several suggestions on how best to promote it to the broader farming community.

### 4.1. Farmer’s Relationship with Cats

Farmers discussed a range of relationship types with cats on the farm, with some comparing them to livestock and others stating they loved them. Most farmers discussed having positive interactions with the cats and that being around the cats positively impacted their mood, which is evidenced by one farmer stating, ‘when you have a bad day and cat comes gives you a rub on the leg it’s hard to be cranky with them’. The benefits of human–animal interactions have been well documented in companion animals [62,63,64,65]. However, many studies often highlight the benefits of companion animals to those already experiencing poor mental health or negative emotions or moods. Farmers are documented to experience higher levels of stress and poorer mental health than other members of the population [40,66,67,68], suggesting that farmers are likely to experience emotional and physical benefits from interactions with cats. There is a lack of literature on the impact of farmers’ relationships with animals on farmers’ wellbeing, with most studies focusing on the impact of farmer–animal relationships and its impact on livestock welfare and production yield [69]. Further research needs to be conducted into the relationship farmers have with working animals and how it impacts farmers’ wellbeing as well as the animal’s welfare. However, our study indicated that interactions with working cats generally had a positive impact on the farmers’ emotions, suggesting a positive relationship or bond between dairy farmers and their working cats. Additionally, farmers discussed providing care for cats, and this is documented to benefit cat owners’ moods or emotions, and pet owners who provide higher levels of care often have stronger bonds with their pets [70,71]. The results of our study suggest that dairy farmers benefited from providing care for cats, and those providing higher levels of care, such as veterinary care, perhaps had a stronger bond with the cats on their farm. Further research investigating farmers’ attachment to their working cats and the contributory psychosocial elements is warranted. Regardless, our study highlights that the relationship or bond dairy farmers have with working cats is viewed by most dairy farmers as beneficial and should be considered when implementing strategies for rural cat management.

### 4.2. Cat Behavior and Welfare

Farmers indicated an improvement in cat behavior and welfare after the cats were sterilized in the program. The positive impact of sterilization on cat welfare is well documented, as it improves body condition and reduces agonistic behaviors, therefore, reducing injuries [44,50,72], which is consistent with our findings. Sterilization has also been shown to reduce nuisance behaviors, such as spraying, fighting, yowling, and unwanted litters [44,72,73,74], again consistent with the findings in our study. Farmers indicated that, prior to sterilization, these behaviors created an unpleasant work environment. Whilst farms tend to be ‘dirty’ workplaces, dairy farms are primary producers, and the presence of feces and deceased animals can create an unhygienic work environment.

Farmers also discussed instances of cat infanticide, which is a rare behavior in domestic cats and has only been documented in rural areas where multiple entire male cats inhabit the area, which leads to female cats becoming aggressive and being injured during the agonistic encounter between female and male cats [75,76,77]. Our study suggests that this behavior may not be as uncommon in domestic cats as initially thought, but sterilization reduces the incidences of infanticide, leading to a more pleasant environment for farmers and better welfare outcomes for cats. That there was a positive relationship between the initial number of cats and the total number euthanized, and that there was a positive association between the total percentage of cats euthanized and the percentage euthanized due to excess numbers, would suggest that early implementation of sterilization programs to limit cat numbers would benefit cat welfare. Only farms with initial cat numbers greater than 50 had more than 2 cats euthanized for welfare reasons. Similarly, with one exception, only farms with initial cat numbers greater than 50 cats had healthy cats euthanized because they were in excess. Because killing cats was unpalatable to farmers and has been shown to negatively impact the mental health of veterinarians and other animal care professionals [35,36], it is recommended that, where possible, sterilization should be used to limit cat numbers before they exceed the number required. However, where there are existing healthy cats in excess of farmer needs, rather than ask farmers to kill cats before the trapping team arrives or veterinarians being required to kill healthy cats, these should be sterilized, returned to the farm, and then be advertised through farming channels and relocated over time to other farms requiring working cats that are sterilized.

Farmers also discussed cats becoming more sociable, stating that ‘[the cats] are much friendlier’ and ‘a lot better natured’. This has been previously documented in the literature [5,78]. Improved relationships between farmers and their cats were commonly reported, as well as the positive impact this had on their emotional wellbeing. Therefore, assisting dairy farmers in sterilizing cats resulted in improved cat behavior and welfare, reduced the number of cat deaths on farms, improved the work environment for farmers, and improved farmer wellbeing.

### 4.3. Cats Impact on the Environment

Whilst domestic cats have been shown to predate mainly on rodents, and prey less frequently on birds when in rural areas than in urban areas [79], farmers were still concerned about the impact the cats had on wildlife, particularly bird species. Prior to sterilization, sightings of hunting behaviors and dead wildlife were reported. After cats were sterilized, farmers perceived a reduction in the cats’ impact on wildlife. A reduction in the cat population is likely to have reduced their impact on wildlife, not only because there are fewer cats predating but also because fewer cats require less food, so the amount provided by the farmer was more likely to be sufficient, reducing the likelihood of cats moving into the environment to supplement their diet. Additionally, the removal of kittens for adoption and preventing unwanted litters from being produced further reduces the rate of predation because young cats have been shown to be more likely to hunt than older cats [28,80]. Moreover, sterilization has been indicated in some studies to reduce home ranges [46,47], which is consistent with our results, where dairy farmers perceived cats to be more ‘homely’, further contributing to the reduction in hunting behaviors and dead wildlife reported in our study.

Because, juvenile cats are the primary shedders of *Toxoplasmosis gondii (T. gondii)* oocysts, reducing the number of juvenile cats will decrease the likelihood of infections in wildlife [81,82]. High levels of cat feces may also increase the risks of *T. gondii* infections, and whilst the health risks to cattle are minimal [83], humans and other animals are at risk of being infected. Juvenile cats defecate more frequently and closer to farm buildings than adult cats, so reducing the number of kittens and juvenile cats on farms through sterilization and adoption is likely to reduce the risk of *T. gondii* infection [81,82,83,84].

Our assistive One Welfare approach to managing these cats using care measures, such as sterilization and rehoming of unwanted kittens, was reported to stabilize and reduce cat populations, decrease hunting behaviors, and mitigate negative impacts on wildlife.

### 4.4. Management Options

Farmers described having an absence of options for the management of their working cat population prior to the sterilization program. Due to a lack of resources and support, lethal management often felt like the only option for farmers. This not only has a negative impact on the people conducting the culling; it is also usually ineffective long-term at reducing free-roaming cat populations. This is because the intensity and duration of culling are typically insufficient to overcome the fecundity of cats, with 40% to 60% of the population requiring to be culled every 6 months for 10 years to have a lasting impact [85,86,87,88]. As farms often provide an abundant source of food and shelter for free-roaming cats, culling the population is likely to lead to an increase in prey species such as rats and mice. Consequently, an increase in the survival of kittens, and the high fecundity, necessitates continuous culling by farmers in an attempt to keep the population down.

Using lethal methods to control the cat population on their farms was neither something farmers liked nor wanted to undertake. They indicated feeling negatively toward this management strategy, stating that it was ‘difficult’ and ‘we didn’t really wanna do that’. Culling livestock has been shown to have a detrimental impact on farmers’ mental health [89,90], and the culling of free-roaming cats has been shown to have a detrimental impact on those caring for them [86]. Farmers discussed a range of relationship types between themselves and the cats, some viewing them as livestock and others indicating having a strong bond. Our results, combined with findings from the literature [86,89,90], indicate that, regardless of the level of relationship, using lethal methods of cat management can have a negative impact on human wellbeing. Lethal cat management strategies currently available to farmers fail to encompass a One Welfare approach. They have harmful consequences for the welfare of cats, fail to protect wildlife because they are not sustained with sufficient magnitude to substantially reduce cat populations, and negatively impact dairy farmers’ wellbeing.

Some farmers discussed sterilizing a small number of cats each year to try to control the cat population, but they were reluctant to use lethal methods, as they valued having the cats on the farm. Sterilization at low levels is an ineffective method of controlling cat populations [87]. This may make farmers feel like their efforts are futile if they do not have the resources to sterilize cats at a sufficiently high rate. Additionally, farmers discussed barriers to sterilization, such as cost and difficulty in catching cats. Consistent with our findings, cost has previously been indicated as one of the main barriers to sterilization [72], with household income being one of the main predictors of whether or not a pet is sterilized [71,91]. These barriers, coupled with a reluctance to use lethal management methods, have led some farmers to do nothing to manage the cat population. Thus, poor welfare outcomes for cats and farmers, and negative impacts on wildlife populations, prevail. Supporting dairy farmers to control cats effectively, with an approach that supports them, is instrumental to reducing farm-cat populations and their potential impacts on wildlife. Therefore, providing funding for these programs, which encompass the One Welfare philosophy, is crucial to reducing the risk to wildlife and improving human and animal welfare.

An additional confounding issue relayed by some farmers was the negative attitudes held toward animal welfare agencies, specifically that this was a barrier to reaching out for help regarding working cat populations and raised skepticism about the sterilization program when they were first approached. There is very little research investigating farmers’ perceptions of animal welfare agencies, although they have been indicated to have a distrust of government agencies [92]. Our results suggest that there is a disconnect between the farming community and animal welfare agencies, similar to that reported between cat owners and semi-owners and local government Animal Management Officers [93]. Public engagement has been suggested to increase trust in government agencies [94], which could be applied to farmer’s distrust of animal welfare agencies. This is supported by our findings, whereby farmers discussed initially having a negative attitude toward the program; however, after the program coordinator engaged and built a relationship with the farmers, they were more willing to reach out for help.

### 4.5. Views on Working Cat Sterilization Program

When discussing the sterilization program, farmers were positive about the process and outcomes, stating that the program was ‘an outstanding success’ and ‘so effective’. After the sterilization program to reduce kitten births, the adoption of existing kittens, and the euthanasia of sick and unwanted cats, the average cat population on farms almost halved (47.2%), which is not dissimilar to the results found in other studies (55% in [95] and 48% in [96]). Thus, over time, sterilization programs are successful in reducing free-roaming cat populations [28,92,94]. Additionally, building rapport between the program coordinator and farmers was critical to population management, as it facilitated high levels of reporting of entire cats and, in turn, sterilization of all remaining working cats. The ongoing success of sterilization programs in reducing free-roaming cat populations relies on continued communication and surveillance of the cats by farmers so that sterilization of new, entire immigrant cats can be arranged when they are identified on site [97]. Farmers indicated that having someone to help catch and organize sterilization for the cats was essential, as they did not have the time nor the resources to do that themselves. Any necessity for dairy farmers to be ‘off-farm’ will act as a barrier; therefore, having support for this process is likely to lead to higher sterilization rates for working cats. Farmers view working cats as an essential part of the farm and will continue to have cats in and around farm buildings. Therefore, supporting farmers to seek help and report new cats for sterilization around farm buildings is crucial to managing working cat populations, decreasing their negative impact on wildlife, ensuring food safety, and ultimately improving cat and human welfare.

### 4.6. Impact on Dairy Farmers

When discussing their feelings before and after participating in the sterilization program, there was a substantial difference in the language farmers used when discussing the situation and their feelings. Farmers used negative language, such as ‘stressful’, ‘heartbreaking’, and ‘embarrassed’, before becoming involved in the sterilization program. Contrastingly, when considering their situation after utilizing the sterilization program, farmers used positive language, such as ‘grateful’, ‘relief’, and ‘warm and fuzzy’.

The World Health Organization lists positive and negative emotions as a facet of the psychological domain of quality of life [98]. The move from negative language to positive language to describe emotions suggests that the sterilization program had a positive impact on farmers’ psychological wellbeing and quality of life. Moreover, prior to the sterilization of the working cats, farmers discussed feelings of having no control but wanting to do the right thing for the cats and not having the knowledge or resources to do so. These feelings could result in moral distress (when one knows the right course of action but is unable to take it due to obstacles and constraints) [99]. This can lead to several negative psychological and physical impacts [100]. Interestingly, after participating in the working cat sterilization program, farmers relayed feelings of personal agency, achievement, and pride, with farmers stating, ‘I’m actually sort of quite proud of [the cats]’ and ‘you feel like you’ve accomplished something’. These feelings have been linked to positive wellbeing outcomes, reducing the risk of moral distress, and have been shown to drive behavioral change, an essential element in the implementation and success of sterilization programs [97,98,101,102]. Furthermore, improvement in farmers’ work environment as a result of fewer cats is likely to positively impact their quality of life and contribute to their overall wellbeing [98,101]. With farmers’ increased risk for poor mental health and suicide in comparison to the general population [37,38,39], it is pertinent to support dairy farmers in taking control of their working cat populations in a manner that does not risk the human–animal relationship. An assistive approach is likely to improve dairy farmers’ quality of life and create positive emotions, which may help reduce risk factors for poor mental health and ill-being.

### 4.7. Views on Barn Cat Program

In Australia, many cats that are admitted into municipal pounds and shelters show fearful, timid, or aggressive behavior. These cats may be deemed feral and euthanized within 24 h, without being given time to adapt to their new environment [103]. Most stray cats who appear unsocialized when first admitted into shelters or municipal pounds are unidentified outdoor owned or semi-owned cats (cats that are cared for by people who do not perceive ownership) [104,105]. Cats that are fearful, timid, or shy are at high risk of euthanasia in a shelter or municipal pound. In Barn or Working Cat Programs, healthy cats not suitably socialized for adoption as family pets are rehomed on farms, stables, or businesses for rodent control. They are increasingly being utilized by shelters to reduce the number of healthy cats euthanized. There are a number of programs like this across North America and the UK [13,14,15,16,17,18,19], and they are starting to be embraced in Australia [20,106,107,108].

Farmers expressed concern about replenishing their cats after natural attrition had reduced numbers, stating that they need to have cats on the farm. The first part of our study documented the essential role cats play on farms in reducing damage to infrastructure caused by rodents and how dairy farmers preferred cats to rodenticide baits as they perceived cats as a cheaper, more efficient, and safer option for people, working dogs, pets, and the environment [12]. A Barn Cat Program would allow farmers to replenish their cat population with cats that have been sterilized and vaccinated, eliminating any risk of unwanted litters leading to a population rebound. Additionally, this would help mitigate capacity issues in shelters and municipal pounds and decrease the number of healthy cats euthanized. All interviewed farmers conveyed positivity around the prospect of a Barn Cat Program, using words such as ‘great’ and ‘good’ to describe the idea of such a program. They also suggested that a mechanism should be in place to ensure farmers take responsibility for the cats and look after them, perhaps through a small fee or an annual check-in. Whilst an annual check-in is not feasible due to constraints on resources, an affordable fee for adoption to cover some sterilization and vaccination costs would benefit the program by recouping some costs. Potentially, it might also increase the value of the cats to farmers and increase the incentive for farmers to provide higher levels of care, which would improve the cats’ welfare. Additionally, one farmer indicated a preference for semi-domesticated cats, as they were easier to keep on the farm. Cats in shelters and municipal pounds that are difficult to adopt because of timid, shy, and fearful behavior are mostly unidentified stray cats, which are presumed to be free-roaming owned or semi-owned cats. Because they were often the cause of nuisance behavior complaints, they have been living around people, and it would be expected that those being placed on farms would be somewhat habituated or socialized to humans [104]. Our results indicate that dairy farmers support a Barn Cat Program. Regardless, there are several Barn Cat Programs operating around the world, but very little literature on their success is available. Further research on the key success factors of Barn Cat Programs and the resources and support required for Australian farmers to participate in such programs is recommended. This knowledge would benefit the wider implementation of these programs and facilitate their success.

When farmers were asked how a Barn Cat Program might best be promoted to farmers, word of mouth was the most common response. Specifically, through farming channels. Farmer-to-farmer information channels are commonly used and are very important sources of information and peer support in rural areas, as are farmer groups (e.g., Dairy Australia) [109,110]. Effective engagement that creates awareness, understanding, and trust is imperative to the successful promotion of animal-related programs. For example, when members of the public were provided with information on the benefits of sterilization compared to traditional trap–adopt or kill programs for the management of urban cats, support for the program increased [23], indicating that the provision of information that improves understanding increases support for programs, which is consistent with the responses in our study. Further research into the benefits of cats on farms and the outcomes of Barn Cat Programs would be beneficial, and this information could then be used when implementing a Barn Cat Program.

### 4.8. Limitations

The limitations of this study have been previously described in a companion paper, which focused on establishing the value of working cats to dairy farmers and their view on the Australian Tax Office classifying cats on farms as working cats [12]. However, as this study had differing aims, some of the limitations also differ. Specifically, the small sample size of this study could be seen as limiting, but the rich lived experience of the interviewees allowed substantial insight and an in-depth understanding of the relationship dairy farmers have with the working cats on their farms, including their experiences before and after participation in a free working cat sterilization program and their views on a Barn Cat Program. The smaller sample size was necessary to gather contextual and detailed data within the project’s timeframe and financial constraints. Additionally, by targeting farmers who had participated in a free working cat sterilization program, participants were limited to exclusively dairy farmers. However, to participate in the study, farmers must have participated in one of two working cat sterilization programs, and therefore, our population sample size is an appropriate representation of this small population, because farmers from all but one of the 10 farms participated in the study. Previous qualitative research of a similar nature has used a small sample size to gain an in-depth and rich understanding of a topic [5,86]. We hope our study can be used as a basis for future research to build upon. Additionally, the nature of in-depth interviews means that there is a potential for there to be self-reporting bias, which could impact the accuracy and reliability of the data. We recognize that participants may have altered their responses due to social desirability bias and could have potentially been influenced by the interviewer’s behavior [111]. To mitigate this, the interviewer was trained to adopt a neutral, nonjudgemental approach, and the confidentiality and anonymity of responses were emphasized. Nonetheless, we acknowledge this as a limitation of our study.

## 5. Conclusions

Current cat management options for farmers do not consider the relationships and value that cats bring to dairy farmers and are focused on the use of lethal cat management methods, which farmers find undesirable. This has a negative impact on farmers and creates a lack of effective intervention, leading to poor outcomes for cats and wildlife. Our study of a working cat sterilization program, which successfully reduced cat populations on participating dairy farms, improved cat welfare and increased farmer wellbeing, demonstrates the interconnectedness of human wellbeing, animal welfare, and the environment. Furthermore, it improved the relationship between animal welfare agencies and farmers, leading to a higher rate of reporting of cats requiring sterilization, and eliminated the need for lethal control methods. This, in turn, reduced the risk of negative mental health impacts on farmers, because it supported farmers in taking control of their working cat populations in a manner that did not damage the human–animal relationship. This method, which aligns with a One Welfare approach, is more acceptable to dairy farmers than current management options and is, therefore, more effective in reducing working cat populations and their impacts on wildlife, whilst positively impacting farmers’ wellbeing and cat welfare.

The dairy farmers in our study viewed cats as a necessity on their farms, with this being their preferred method of rodent control for safety, environmental, and companionship reasons. The implementation of sterilization programs alongside Barn Cat Programs would reduce uncontrolled working cat populations whilst allowing farmers to continue to have cats assisting with vermin control and providing companionship on farms. Prior to and throughout implementation, farmers should be consulted, and their suggestions should be considered to ensure ongoing trust between farmers and animal welfare agencies to ensure the success of these programs. Given the essential role working cats play for dairy farmers alongside the environmental concerns surrounding unmanaged populations of free-roaming cats, we strongly recommend funding be provided for both sterilization programs and Barn Cat Programs. All relevant stakeholders including federal and state governments, relevant industry groups, animal welfare agencies, and conservation agencies should be engaged to support this initiative. Once the farm-cat populations are controlled by initial externally funded programs, if veterinary costs are tax-deductible for working cats, then farmers would likely be able to prevent the population from rebounding [12]. The funding for the implementation of such programs would help to effectively control working cat populations in a way that appeals to farmers and reduces impacts on the environment and wildlife, whilst benefiting human wellbeing and animal welfare. Additionally, future research should extend to other farmer groups, such as grain farmers or those with expensive machinery, as our study indicates that these may be the farming groups most impacted by rodent overpopulation and would, therefore, benefit from these programs.

A One Welfare approach acknowledges the interconnectedness of people, animals, and their environments. This approach appreciates the value placed on working cats by those who interact with them and considers the potential impacts on wildlife and the environment. Incorporating these concepts and the framework in which they sit into domestic cat management strategies will result in successful reductions in cat populations and improvements in cat and wildlife welfare and human wellbeing.

## Data Availability

Most relevant data are reproduced in the text.

## Appendix A

**Table A1:** Number and proportion (%) of the initial number of cats on each farm that were sterilized and either returned to the farm or rehomed, or where euthanized, and number and proportion of cats euthanized that were euthanized for welfare reasons or because they were in excess of the numbers required for the farm. Data shown as number (%).

Farm	Initial Number of Cats	Returned	Rehomed	Euthanized	Euthanized for Welfare Reasons	Euthanized Due to Excess Numbers	Final Cat Number	Percentage Change Between Initial and Final Cat Numbers
1	3	3 (100%)	0 (0%)	0 (0%)	0 (0%)	0 (0%)	3	0%
2	57	35 (61.4%)	5 (8.8%)	17 (29.8%)	4 (7.0%)	13 (22.8%)	35	−38.6%
3	175	79 (45.1%)	50 (28.6%)	46 (26.3%)	43 (24.6%)	3 (1.7%)	79	−54.9%
4	22	10 (45.5%)	0 (0%)	12 (54.54%)	2 (9.1%)	10 (45.5%)	10	−54.5%
5	61	33 (54.1%)	5 (8.2%)	23 (37.7%)	13 (21.3%)	10 (16.4%)	33	−45.9%
6	8	8 (100%)	0 (0%)	0 (0%)	0 (0%)	0 (0%)	8	0%
7	31	12 (38.7%)	17 (54.8%)	2 (6.5%)	2 (6.5%)	0 (0%)	12	−61.3%
8	20	12 (60%)	8 (40%)	0 (0%)	0 (0%)	0 (0%)	12	−40%
9	15	15 (100%)	0 (0%)	0 (0%)	0 (0%)	0 (0)	15	0%
**Total**	**392**	**207 (52.8%)**	**85 (21.7%)**	**100 (25.5%)**	**64 (16.3%)**	**36 (9.2%)**	**207**	**−47.2%**
